# Phylogenetic Implications of Mitogenomic Sequences and Gene Rearrangements of Scale Insects (Hemiptera, Coccoidea)

**DOI:** 10.3390/insects14030257

**Published:** 2023-03-05

**Authors:** Han Xu, Xiaochen Liu, Pei Wang, Hu Li, San-an Wu

**Affiliations:** 1 The Key Laboratory for Silviculture and Conservation of Ministry of Education, Beijing Forestry University, Beijing 100083, China; 2 Department of Entomology, College of Plant Protection, China Agricultural University, Beijing 100193, China; 3 Key Laboratory of Molluscan Quarantine and Identification of GACC, Fuzhou Customs District, Fuzhou 350001, China

**Keywords:** Coccoidea, gene order, molecular phylogeny

## Abstract

**Simple Summary:**

Coccoidea (scale insects) are sap-sucking hemipterous insects with a high diversity of species. They exhibit dramatically variable appearance and sexual dimorphism and are also closely related to human beings as important economic pests and resource insects. However, we know very little about the phylogenetic relationship within Coccoidea. We reconstructed that relationship among five coccoid families (e.g., Aclerdidae, Cerococcidae, Coccidae, Eriococcidae, and Kerriidae) based on mitogenomes. Aclerdidae and Coccidae were recovered as the sister group, successively sister to Cerococcidae, Kerriidae, and Eriococcidae. In addition, there were gene rearrangements occurring in all mitogenomes of coccoid species studied here. The novel gene rearrangement *ND6-trnP* and *trnI-ND2-trnY* supported the monophyly of Coccoidea and the sister relationship of Aclerdidae and Coccidae.

**Abstract:**

Coccoidea (scale insects) are important plant parasites with high diversity of species. However, the phylogenetic relationship within Coccoidea has not been fully determined. In this study, we sequenced mitogenomes of six species belonging to five coccoid families. With the addition of three previously published mitogenomes, a total of 12 coccoid species were adopted for the phylogenetic reconstruction based on the maximum likelihood and Bayesian inference. The monophyly of Coccoidea was recovered and Aclerdidae and Coccidae were recovered as the sister group, successively sister to Cerococcidae, Kerriidae, and Eriococcidae. In addition, there were gene rearrangements occurring in all mitogenomes of coccoid species studied here. The novel gene rearrangement *ND6-trnP* and *trnI-ND2-trnY* supported the monophyly of Coccoidea and the sister relationship of Aclerdidae and Coccidae. This implies that data from the mitogenome can provide new insight for clarifying the deeper level of phylogenetic relationship within Coccoidea.

## 1. Introduction

Scale insects (Coccoidea) are small and sap-sucking hemipterans and closely related to Aphidoidea (aphids), Aleyrodoidea (whiteflies), and Psylloidea (jumping plant lice), belonging to the suborder Sternorrhyncha. Currently, there are more than 8000 species described in all zoogeographical fauna, belonging to 36 extant families [1]. Interestingly, they are extremely sexually dimorphic, adult females are paedomorphic and highly reduced while males have a more “typical” insect appearance and display complete metamorphosis [2,3].

The current classification of Coccoidea is mainly based on the morphology of adult females. However, the morphological features of adult females of coccoids have undergone significant reduction, providing very limited phylogenetic information; therefore, little is known about the relationship among coccoid families and the monophyly of some families remains verified, especially Eriococcidae [2]. Subsequently, Cook et al. [4] and Gullan and Cook [5] adopted a nuclear RNA gene (SSU rRNA or *18S*) to infer the phylogenetic relationship among the coccoid families. These studies verified the monophyly of various families but Eriococcidae and recovered the sister relationship between Coccidae and Kerriidae without Aclerdidae involved. The morphology of adult males was believed to improve our understanding of evolutionary relationships among coccoid families [6,7,8,9]. Hodgson and Hardy [10] estimated the phylogeny of Coccoidea based on the morphological characters of adult males, supporting the sister relationship between Aclerdidae and Coccidae. Cerococcidae was recovered as a sister group with the clade of Aclerdidae and Coccidae. Vea and Grimaldi [11] reconstructed the relationship at the family level within Coccoidea combining the morphology and gene fragments (e.g., *18S*, *28S*, and *EF-1a*), also indicating the closest relationship between Coccidae and Aclerdidae. However, Kerriidae was found to have a closer affinity with Cerococcidae, inconsistent with previous results based on either several molecular markers or morphology. Furthermore, Eriococcidae was consistently found to be paraphyletic by the above studies both based on the molecular and morphological data [4,5,10,11]. Liu et al. also reconstructed the phylogenetic relationship based on the ultraconserved element (UCE) [12]. However, Diaspididae was found to be closer to Eriococcidae, represented by *Acanthococcus lagerstroemiae*, this was contrary to the morphology of male adults [10]. Accordingly, the relationship within Coccoidea was not fully understood using morphological or limited gene fragments [5], therefore further study is necessary. 

The mitogenome is a circular and double-stranded DNA molecule. It has been widely adopted as a powerful molecular marker for phylogenetic and evolutionary analysis in various insect groups, especially high taxonomic levels, because of the relatively high evolutionary rate and rare recombination [13,14,15]. Moreover, there are gene rearrangements frequently occurring in the mitogenomes of insects. This gene structure information is also useful in inferring phylogenetic relationships [16,17,18,19,20]. There have been thirteen coccoid mitogenomes recorded in GenBank [21,22,23,24,25]; however, eight of which were regarded as unverified data (e.g., *Ceroplastes japonicus*, *C. rubens*, *Drosicha corpulenta*, *Ericerus pela*, *Nipponaclerda biwakoensis*, *Planococcus citri*, *Saissetia coffeae*, and *Unaspis yanonensis*). Moreover, the putative “*P. citri* and *U. yanonensis*” mitogenomes reported by Liu et al. [23] were lately considered not to originate from Coccoidea but from parasitic wasps in the Chalcidoidea [26]. Recently, there were gene rearrangements occurring in six valid coccoid mitogenomes and the phylogenetic results based on the mitogenomic sequence frequently found the monophyly of Coccoidea [24,25], thus indicating that the mitogenomes may have the potential in determining the phylogenetic relationship within Coccoidea. In this study, we sequenced four species from Cerococcidae, Eriococcidae, and Kerriidae, and re-sequenced two from Aclerdidae and Coccidae based on the next-generation sequencing method.

## 2. Materials and Methods

### 2.1. Sampling and Genomic DNA Extraction

Six species belonging to six families of Coccoidea were collected in China and Australia and were preserved in 95% ethanol (Tianjin Huihang Chemical Technology Co, Tianjin, China) under −20 °C at the Department of Forestry Protection, Beijing Forestry University (Table 1). The total genomic DNA was extracted from the whole body using QIAamp DNA Micro Kit following the manufacturer’s instructions.

### 2.2. Mitogenome Sequencing and Assembly

We sequenced six mitogenomes using a high-throughput sequencing platform with Illumina Hiseq 2500 at Berry Genomics (Beijing, China) with 6× sequencing depth and with 250 bp paired-end sequencing reads. An Illumina TruSeq library with single species was constructed from total genomic DNA with an average insert size of 450 bp. After removing adapters and low-quality reads, high-quality reads were used in de novo assembly with IDBA-UD [27]. Assemblies with IDBA-UD used a similarity threshold of 98% and minimum and maximum k values of 80 and 240 bp, respectively.

Additionally, *COI* and *srRNA*, two molecular fragments from mitogenomes, were amplified as bait sequences by standard PCR reactions using primers designed with reference [28], and then were adopted to identify the mitogenome assemblies. The mitogenomic sequences were identified by Geneious 10.1.3 (http://www.geneious.com/, accessed on 15 March 2019) with a BLASTn search [29] against the reference of bait sequences. Only hits with a 100% pairwise identity were regarded as successful identification. The identified mitogenomic sequences were manually checked in Geneious 10.1.3 for identical or near-identical overlapping terminal regions and were circularized where possible.

### 2.3. Gene Annotation and Bioinformatic Analysis

The annotation of protein-coding genes (PCGs) was implemented with MITOs WebServer [30] and, subsequently, the accurate position was further determined in MEGA v6.06 [31].

The tRNA genes were detected by MITOs WebServer, ARWEN [32], and the manual method with the reference of other hemipteran species (see the Supplementary Materials for detailed annotation information). The boundaries of rRNAs were determined by the upstream and downstream of tRNAs, and by the alignment with the homologous genes of other hemipteran species. The base composition, codon usages of PCGs, and relative synonymous codon usage (RSCU) values were calculated by MEGA v6.06. The number of synonymous substitutions per synonymous site (Ks), the number of nonsynonymous substitutions per nonsynonymous site (Ka), the effective number of codons (ENC), and the codon bias index (CBI) for PCGs were calculated by the DnaSP 5.0 software [33].

### 2.4. Phylogenetic Analysis

There were 40 species included in the phylogenetic analysis, including 12 coccoid species and 24 species of Sternorrhyncha with other four species of Auchenorrhyncha as outgroups (Table 2). A total of 13 PCGs and two rRNAs were merged into a dataset. Because of unverified data and incomplete numbers of PCG, five coccoid species (e.g., *Ceroplastes rubens*, *Drosicha corpulenta*, *Ericerus pela*, *Planococcus citri*, and *Unaspis yanonensis*), were excluded from this study.

The heterogeneity of sequence divergence within the dataset was evaluated by AliGROOVE with the default settings. In addition, the indels in the dataset were scored by AliGROOVE as ambiguities.

The workflow of the data matrix and phylogenetic analysis was implemented in PHYLOSUITE v1.2.2 [34]. The phylogenetic relationship was reconstructed with the maximum likelihood (ML) method and Bayesian inference (BI). The sequences of PCGs were aligned in MAFFT [35] with the codon alignment model and G-INS-i (accurate) strategy. The nucleotide sequences of rRNAs were aligned in MAFFT with the normal alignment model and G-INS-i (accurate) strategy. Then, the poorly aligned sites of PCGs and rRNAs were removed by Gblocks [36] with the default parameter settings. The modified sequences of PCGs and rRNAs were eventually concatenated in PHYLOSUITE v1.2.2.

The optimal partitioning schemes for ML analysis were calculated by PartitionFinder 2 [37] with default settings. The best model was automatically determined by IQ-TREE 1.6.8 [38] and maximum likelihood phylogenies were inferred using IQ-TREE under the Edge-linked partition model for 8000 ultrafast [39] bootstraps. In addition, the result from AliGROOVE analysis showed a high degree of compositional heterogeneity (Figure S1), which can mislead the reconstruction of the phylogenetic tree [40]. Therefore, the Bayesian tree was reconstructed using Phylobayes MPI 1.4f [41] with the site-heterogeneous model CAT + GTR and a discrete gamma distribution with four rate categories [42,43]. Two independent chains were run each with 10,000 generations, and a consensus tree was calculated with a burn-in of 7500, taking one every 10 trees, up to the end of each chain.

### 2.5. Gene Rearrangement Analysis

The gene rearrangements of coccoid mitogenomes were analyzed by the CREx program [44], employing the common interval measurement with that of *Drosophila yakuba* as the reference.

## 3. Results

### 3.1. General Features and Nucleotide Composition

**Figure 1:**
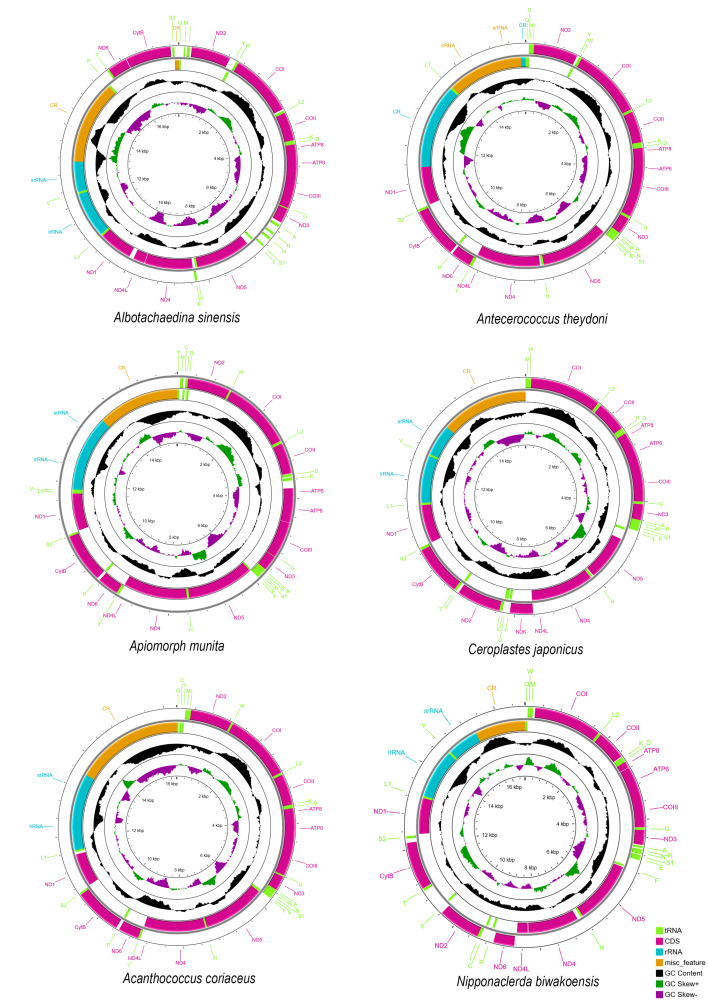
Circular maps of the mitogenomes of Coccoidea.

The length of six mitogenomes of Coccoidea ranged from 15,529 bp in *Ceroplastes japonicus* to 17,405 bp in *Albotachaedina sinensis* (Figure 1). The mitogenomes of *Ap. munita*, *C. japonicus*, and *N. biwakoensis* contain 37 typical genes. However, only 36 genes were detected in those of the other three species, of which *trnV* was not in *Antecerococcus theydoni* and *Acanthococcus coriaceus,* and *trnC* was not in *Al. sinensis*. These coccoid mitogenomes all have high A + T content ranging from 81% in *N. biwakoensis* to 90% in *Al. sinensis*, consistent with the strong bias toward A + T in the findings that *Aclerda takahashii*, *D. koreanus*, and *S. coffeae* previously published [22,24,25].

### 3.2. Protein-Coding Genes

**Figure 2:**
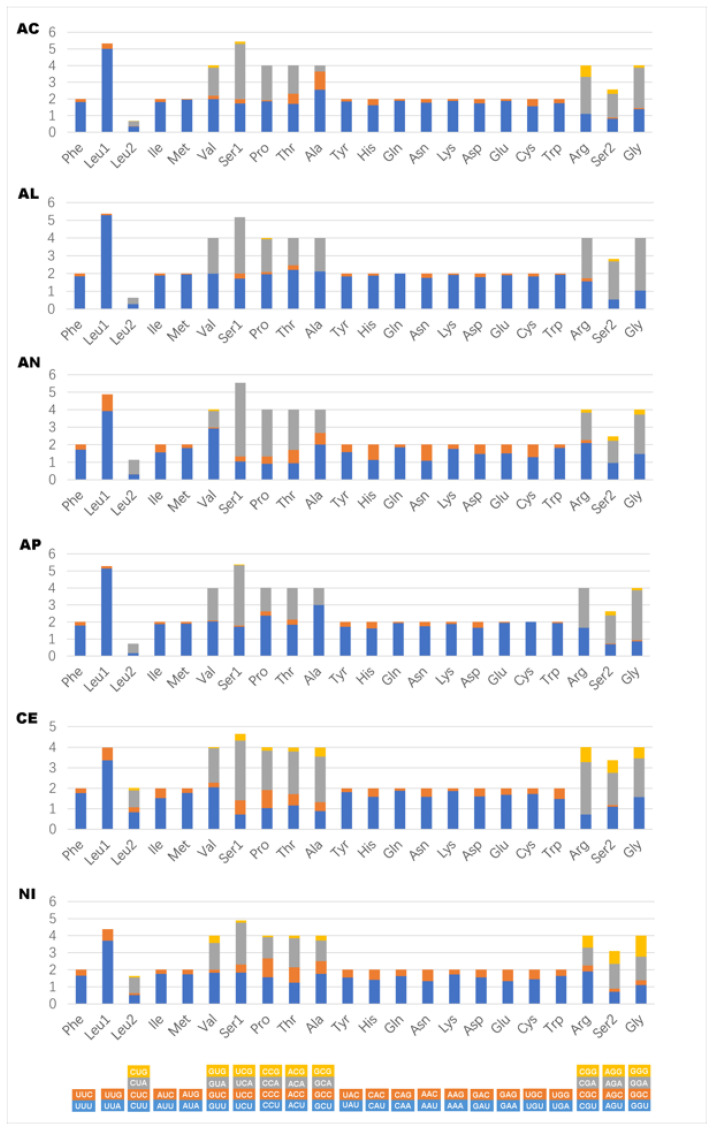
The relative synonymous codon usage (RSCU) in the mitogenomes of Coccoidea. AC, *Acanthococcus coriaceus*; AL, *Albotachaedina sinensis*; AN, *Antecerococcus theydoni*; AP, *Apiomorpha munita*; CE, *Ceroplastes japonicus*; NI, *Nipponaclerda biwakoensis*.

All 13 PCGs were detected in mitogenomes of Coccoidea newly sequenced here, the length of which ranged from 10,500 bp in *Acanthococcus coriaceus* to 10,641 bp in *N. biwakoensis*. The A + T content in PCGs in six newly sequenced coccoid species was high, ranging from 81% in Aclerdidae represented by *N. biwakoensis* to 89% in Kerriidae represented by *Al. sinensis*. In addition, the relative synonymous codon usage (RSCU) of coccoid mitogenomes was examined and shown in Figure 2. The codon ended with A or T was preferred for each amino acid in coccoid mitogenomes newly sequenced here, this is consistent with the strong bias towards A + T content of PCGs in coccoid mitogenomes previously published [22,24]. All PCGs in mitogenomes of Coccoidea studied here initiate with the typical start codon ATN and all terminate with the stop codon TAA or TAG, except for the *COII* in *C. japonicus* and *ND4* in *Acanthococcus coriaceus* with a T residue as the stop codon. 

**Figure 3:**
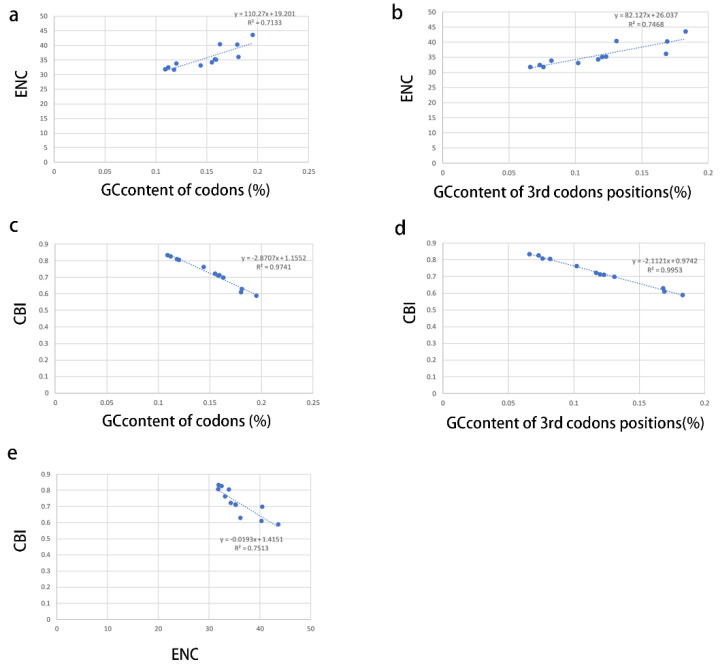
Evaluation of codon bias across 12 coccoid mitogenomes. ENC, effective number of codons (out of a maximum of 61); CBI, codon bias index; G + C, GC content of codons; (G + C)3, GC content of 3rd codon positions. (**a**), relevance of GC content of codons to ENC; (**b**), relevance of GC content of 3rd codons positions to ENC; (**c**), relevance of GC content of codons to CBI; (**d**), relevance of GC content of 3rd codons positions to CBI; (**e**), relevance of ENC to CBI.

The correlations among ENC (effective number codon), CBI (codon bias index), GC content of all codons, and GC content of the 3rd codon positions were analyzed to further investigate the codon usage bias (Figure 3a–e). A positive correlation was observed between ENC and GC content of all codons (R^2^ = 0.7133) (Figure 3a) and GC content of the 3rd codon positions (R^2^ = 0.7468) (Figure 3b), whereas a negative correlation was observed between CBI and GC content of all codons (R^2^ = 0.9741) (Figure 3c) and GC content of the 3rd codon positions (R^2^ = 0.9953) (Figure 3d) and ENC (R^2^ = 0.7513) (Figure 3e). These results show that the GC content is significant for the codon usage bias in Coccoidea, consistent with the neutral mutational theories [45,46].

**Figure 4:**
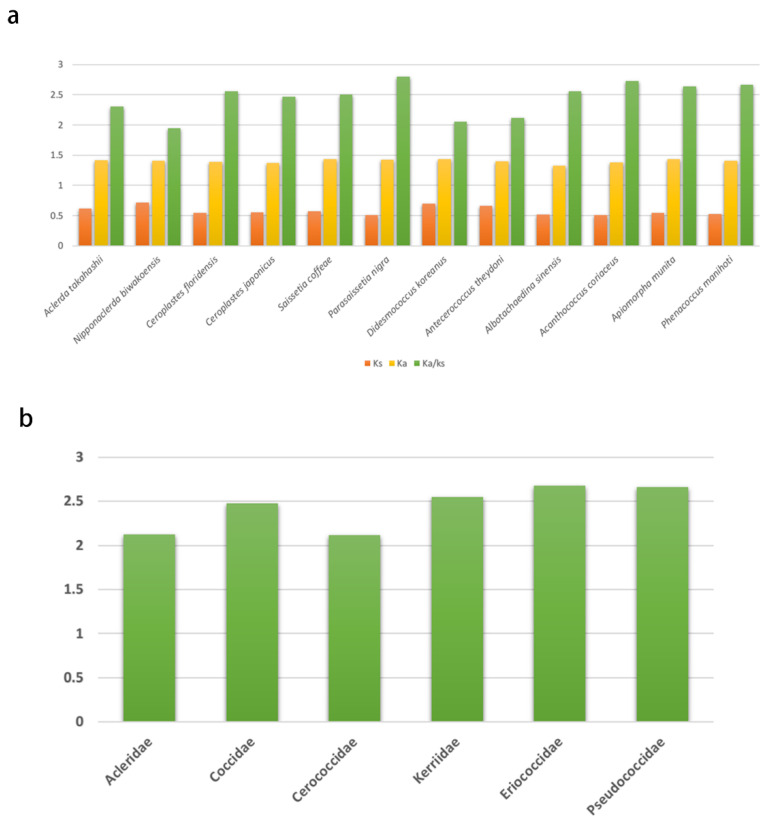
Evolutionary rates of all protein-coding genes (PCGs) in the mitogenomes of 12 coccoid species with *Aphis citricidus* as the reference. The Y-axis provides the substitution rate of mitochondrial PCGs. (**a**) Evolutionary rates of PCGs of each coccoid species; (**b**) average evolutionary rates of PCGs of coccoid families.

The rate of non-synonymous substitutions (Ka), the rate of synonymous substitutions (Ks), and the ratio of Ka/Ks were calculated for PCGs (Figure 4) to diagnose the evolutionary rates of protein-coding genes of coccoid mitogenomes [47,48]. All ratios of Ka/Ks for PCGs of each coccoid species were consistently higher than one (Table S1). The average ratios of Ka/Ks ranged from 2.11 for Cerococcidae to 2.68 for Eriococcidae were consistently higher than one (Figure 4), indicating that all PCGs of coccoid mitogenomes are evolving under positive selection.

### 3.3. Transfer and Ribosomal RNA Genes

The length of tRNA genes in coccoid mitogenomes ranged from 43 bp to 74 bp. There are only a few tRNA genes in each coccoid species studied here that can fold into typical cloverleaf secondary structures (e.g., *trnA*, *trnI*, and *trnL2* in *Al. sinensis*, *trnK*, *trnL2*, and *trnW* in *Antecerococcus theydoni*, *trnK*, *trnL1*, *trnL2*, *trnT*, and *trnV* in *Ap. munita*, *trnK*, *trnL1*, *trnL2*, and *trnM* in *C. japonicus*, *trnF*, *trnK*, and *trnM* in *Acanthococcus coriaceus*, *trnK*, *trnI*, *trnL2*, and trnM in *N. biwakoensis*), while most of the tRNA genes in these six mitogenomes of Aclerdidae, Cerococcidae, Eriococcidae, and Kerriidae were truncated. Similarly, this phenomenon of truncated tRNA genes is also prevalent in mitogenomes of Coccidae reported before [21,22,24].

The rRNA boundaries were determined by alignment with corresponding sequences from Sternorrhyncha previously published. As in other insect mitogenomes, both *lrRNA* and *srRNA* in six coccoid species were encoded on the light strand. The length of *lrRNA* ranged from 1062 bp in *Ap. munita* to 1243 bp in *N. biwakoensis* and that of *srRNA* ranged from 723 bp in *Antecerococcus theydoni* to 792 bp in *N. biwakoensis*. All *lrRNA* of six coccoid species had a high AT content, ranging from 85% in *Antecerococcus theydoni* to 91% in *Ap. munita* and *Al. sinensis*. Likely, all *srRNA* of six coccoid species had a strong bias to AT content, ranging from 83% in *N. biwakoensis* to 92% in *Ap. munita*, *Acanthococcus coriaceus*, and *Al. sinensis*.

### 3.4. Gene Rearrangements Analysis

**Figure 5:**
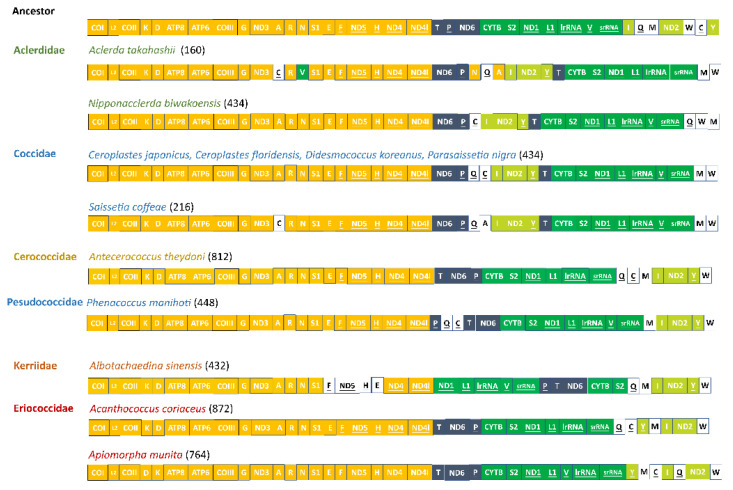
Gene orders in mitogenomes of Coccoidea and the putative ancestor. The boxes with colors indicate relatively conserved gene orders. The genes encoded on the light strand were indicated with underlines. The numbers in parentheses were the value of common intervals.

There were gene rearrangements occurring in six coccoid species sequenced relative to the putative ancestral gene order, including the rearrangement of tRNA genes and protein-coding genes. Based on the result from CREx analysis, the common intervals ranged from 160 in *Aclerda takahashii* to 872 in *Acanthococcus coriaceus* (Figure 5). This means that the highest degree of gene rearrangement relative to ancestral gene order was observed in the *Aclerda takahashii* of the Aclerdidae family, while the lowest degree of that was observed in the *Acanthococcus coriaceus* of the Eriococcidae family. In addition, *C. floridensis*, *C. japonicus*, *D. koreanus*, and *P. nigra* shared the same pattern of gene rearrangement.

Relative to the putative ancestral gene order, there were two relatively conserved gene blocks in mitogenomes of Coccoidea (e.g., *COI-trnL2-COII-trnK-trnD-ATP8-ATP6-COIII-trnG-ND3-trnA-trnR-trnN-trnS1-trnE-trnF-ND5-trnH-ND4-ND4l* and *CYTB-trnS2-ND1-trnL1-lrRNA-trnV-srRNA*). Among the rearranged genes, the *trnP-ND6* ancestral gene block underwent a transposition event to form *ND6-trnP*, a novel gene boundary. This novel gene cluster was frequently present in coccoid species but absent in Pseudococcidae, Kerriidae, and three other sternorrhynchan superfamilies, implying that it may be an apomorphy in Coccoidea. In addition, except for in Eriococcidae, *ND2* was rearranged between *trnI* and *trnY* in Aclerdidae, Coccidae, Cerococcidae, and Kerriidae, forming a novel gene boundary. Additionally, it is notable that *ND2* was translocated between the *ND6* and *CytB* in both Aclerdidae and Coccidae but not in the other three families. 

### 3.5. Phylogenetic Analysis

**Figure 6:**
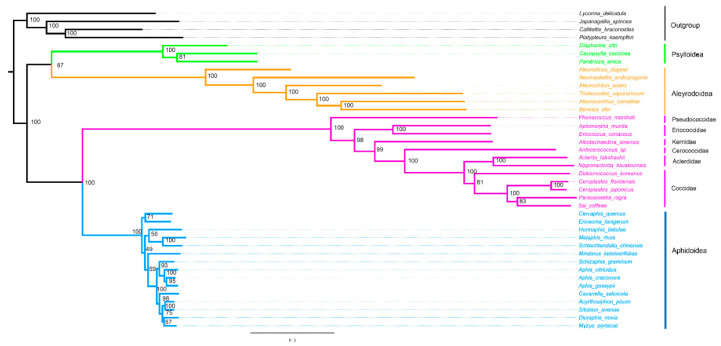
Phylogenetic trees inferred from mitogenomes of Coccoidea under ML method (right). The values at nodes indicate ML bootstrap values.

**Figure 7:**
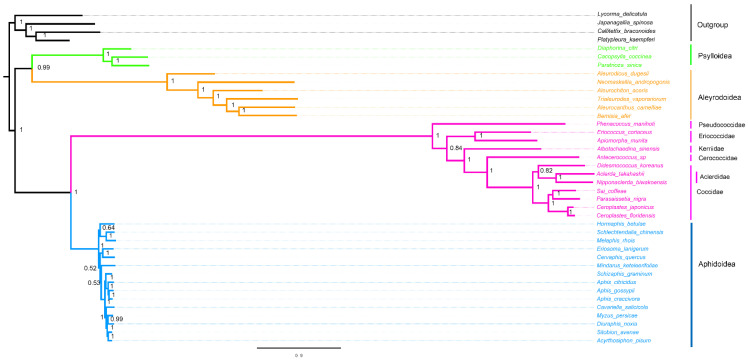
Phylogenetic trees inferred from mitogenomes of Coccoidea under BI. The values at nodes indicate Bayesian posterior probabilities.

The phylogenetic analyses were performed on the nucleotide sequences of 13 PCGs and two rRNAs from mitogenomes of 40 hemipteran species. The phylogenetic relationship generated by BI and ML methods was found (Figure 6 and Figure 7). Two topologies are identical with the exception of the relationship within Aphidoidea and the position of the Aclerdidae coccoid family in this study. The monophyly of Sternorrhyncha was determined (bootstrap value = 100%, posterior possibility = 1) and, within Sternorrhyncha, the monophyly of Coccoidea was supported and other three sternorrhynchan superfamilies (Aleyrodoidea, Aphidoidea, and Psylloidea) were also found as monophyletic groups. Psylloidea was determined to be a sister to Aleyrodoidea with high statistical support. In addition, Coccoidea was grouped with Aphidoidea. Within Coccoidea, a phylogenetic relationship of (Pseudococcidae + (Eriococcidae + (Kerriidae + (Cerococcidae + (Aclerdidae + Coccidae))))) was found with high statistical support.

## 4. Discussion

The relationship within Sternorrhyncha has been controversial. Aleyrodoidea or Psylloidea was found to be at a basal position of Sternorrhyncha based on molecular evidence [43,49]. Aleyrodoidea was grouped with Psylloidea and Coccoidea was sister to Aphidoidea based on morphological characters from both extant and extinct taxa in the latest study [50]. The topology resulting from mitogenomic sequences agreed with the result-based morphological characters.

Within Coccoidea, the Pseudococcidae family has been regarded as a sister to the rest of the neococcoids [5,10,51]. This family was also sister to other coccoid families studied here. The monophyly and systematic position of Eriococcidae has been uncertain. It was believed to be close to Pseudococcidae based on the morphology of adult males [7] and, subsequently, was frequently recovered to be a paraphyly with Dactylopiidae, Beesoniidae, and Stictococcidae based on both morphology and molecular markers [4,5,9]. This family represented by single species *Acanthococcus lagerstroemiae*, belonging to the acanthococcid group defined by the study of Gullan and Cook [5], was grouped with Diaspididae based on the ultraconserved elements (UCEs), excluding the above three families [12]. Two eriococcid species adopted in phylogenetic analysis (e.g., *Acanthococcus coriaceus* and *Apiomorpha munita*) were also members of the acanthococcid group defined by the study of Gullan and Cook [5]. However, the Eriococcidae family represented by the two species above was placed to be sister to other families studied here (e.g., Aclerdidae, Cerococcidae, Coccidae, and Kerriidae). To some extent, this supported the conclusion based on the morphology of adult males [7]. Because the samplings in this study were too limited, the relationship between Eriococcidae with other coccoid families and within this family reflects just a partial portion of the big picture. The relationship between the morphologically peculiar Kerriidae and the other three related families (e.g., Aclerdidae, Cerococcidae, and Coccidae) remains uncertain [52]. It was believed to be closer to Coccidae based on *SSU rRNA* without Aclerdidae and Cerococcidae included [4,5] or closer to Cerococcidae based on the total evidence [11]. Although this family was found to be sister to the clade of Aclerdidae, Cerococcidae, and Coccidae based on the morphology of adult males, this clade was weakly supported with just 0.58 posterior possibilities [10]. In the present analysis, Kerriidae was recovered to be sister to the clade of Aclerdidae, Cerococcidae, and Coccidae in both ML and Bayesian trees with 99% bootstrapping support and 1 posterior possibility, respectively. In addition, Cerococcidae was supported by the result based on the mitogenomes as the sister group to the clade of Aclerdidae and Coccidae. Coccidae was found to be a sister to Aclerdidae in the ML tree with 100% bootstrapping support (Figure 6), consistent with the previous conclusion based on the morphology of adult females and males and DNA sequences [10,11,53,54]. This novel gene boundary *trnI*-*ND2-trnY* supported the phylogenetic result of the grouping of these four families based on the mitogenomic sequences. In addition, the translocation of *ND2* also supported the closest relationship of Aclerdidae and Coccidae as a synapomorphy, as indicated by the result based on the mitogenomic sequences above. Although Coccidae was found to be paraphyletic with the inclusion of Aclerdidae in the Bayesian tree, the closer relationship between both families than other families were supported with 1 posterior possibility (Figure 7). Within Coccidae, the topology of ML and the Bayesian tree was largely similar, except for the Aclerdidae nested in Coccidae in the Bayesian tree. The Ceroplastinae represented by *C. floridensis* and *C. japonicus* had a closer affinity with Coccinae, represented by *P. nigra* and *S. coffeae* in both ML and Bayesian tree, then grouped with Eulecaniinae, represented by *D. koreanus*, corresponding with the topology (Eulecaniinae + (Coccinae B + Ceroplastinae)) [55].

## Figures and Tables

**Table 1:** Collection information of Coccoidea in this study.

Family	Species	Locatlity	Date	Collector	Voucher Number
Cerococcidae	*Antecerococcus theydoni* (Hall, 1935)	Longlin County, Guangxi, China	5 May 2017	Jiangtao Zhang & Ming Zhao	BFU2017050503
Coccidae	*Ceroplastes japonicus* Green, 1921	Mingguang, Anhui, China	6 October 2015	Hu Li	BFU2015100601
Eriococcidae	*Apiomorpha munita* (Schrader, 1863)	Red Rock Gorge, Tuggeranong, Australia	22 January 2012	Xiaobei Wang	BFU2012012212
	*Acanthococcus coriaceus* (Maskell, 1893)	Dutton Park, Queensland, Australia	19 July 2013	Xiaobei Wang	BFU2013071900
Kerriidae	*Albotachaedina sinensis* Zhang, 1992	Tea Horse ancient town, Pu’er, Yunnan, China	19 June 2017	Xu Wang	BFU2017061904
Aclerdidae	*Nipponaclerda biwakoensis* (Kuwana, 1907)	Haidian, Beijing, China	20 January 2018	San-an Wu	BFU2018012001

**Table 2:** Species involved in the phylogenetic analysis.

Suborder	Superfamily/Family	Species	GenBank Accession Number
Auchenorrhyncha	Cercopidae	*Callitettix braconoides*	NC_025497
	Cicadidae	*Platypleura kaempferi*	KY039114
	Cicadellidae	*Japanagallia spinosa*	NC_035685
	Membracidae	*Lycorma delicatula*	NC_012835
Sternorrhyncha	Aleyrodidea	*Aleurocanthus camelliae*	KU761949
		*Aleurochiton aceris*	NC_006160
		*Aleurodicus du* *gesii*	NC_005939
		*Bemisia afer*	NC_024056
		*Neomaskellia andropogonis*	NC_006159
		*Tetraleurodes acaciae*	NC_006292
		*Trialeurodes vaporariorum*	NC_006280
	Aphidoidea	*Acyrthosiphon pisum*	NC_011594
		*Aphis craccivora*	NC_031387
		*Aphis gossypii*	NC_024581
		*Cavariella salicicola*	NC_022682
		*Cervaphis quercus*	NC_024926
		*Diuraphis noxia*	NC_022727
		*Eriosoma lanigerum*	NC_033352
		*Hormaphis betulae*	NC_029495
		*Melaphis rhois*	NC_036065
		*Mindarus keteleerifoliae*	NC_033410
		*Myzus persicae*	NC_029727
		*Schizaphis graminum*	NC_006158
		*Schlechtendalia chinensis*	NC_032386
		*Sitobion avenae*	NC_024683
	Coccoidea	*Acanthococcus coriaceus*	OP351525
		*Aclerda takahashii*	MW8395575
		*Albotachaedina sinensis*	OP351521
		*Antecerococcus theydoni*	OP351522
		*Apiomorpha munita*	OP351523
		*Ceroplastes floridensis*	OK040657
		*Ceroplastes japonicus*	OP351524
		*Didesmococcus koreanus*	MW302211
		*Nipponaclerda biwakoensis*	OP351526
		*Parasaissetia nigra*	OK040656
		*Phenacoccus manihoti*	MZ958983
		*Saissetia coffeae*	MN863803
	Psylloidea	*Cacopsylla coccinea*	NC_027087
		*Diaphorina citri*	NC_030214
		*Paratrioza sinica*	NC_024577

## Data Availability

The data that support the findings of this study are openly available in the National Center for Biotechnology Information at https://www.ncbi.nlm.nih.gov/nuccore (accessed on 1 December 2021), reference numbers OP351521–OP351526.

## Supplementary Materials

The following are available online at https://www.mdpi.com/article/10.3390/insects14030257/s1, Supplementary File S1: mitogenomic annotations of *Acanthococcus coriaceus*; Supplementary File S2: mitogenomic annotations of *Albotachaedina sinensis*; Supplementary File S3: mitogenomic annotations of *Antecerococcus theydoni*; Supplementary File S4: mitogenomic annotations of *Apiomorpha munita*; Supplementary File S5: mitogenomic annotations of *Ceroplastes japonicus*; Supplementary File S6: mitogenomic annotations of *Nipponaclerda biwakoensis*. Figure S1. Heterogeneous sequence divergence within hemipteran mitochondrial genomes in this study. The obtained mean similarity score between sequences was represented by a colored square. The scores were ranging from −1, indicating full random similarity, to +1, non-random similarity. The darker red indicated the higher randomized accordance between pairwise sequence comparisons. Blue indicated the opposite. All taxa names were listed on top and the right-hand side of the matrix. Table S1. Evolutionary rates of all protein-coding genes (PCGs) in the mitogenomes of nine coccoid species with *Aphis citricidus* as the reference.
