# Supplementary material for: Phylogenetic Implications of Mitogenomic Sequences and Gene Rearrangements of Scale Insects (Hemiptera, Coccoidea)
Source: Insects. 2023 Mar 5;14(3):257. doi: 10.3390/insects14030257 (PMC10051623; doi:10.3390/insects14030257)
Supplement: Supplementary file 1 [file insects-14-00257-s001.zip › Figure_S1.pdf]

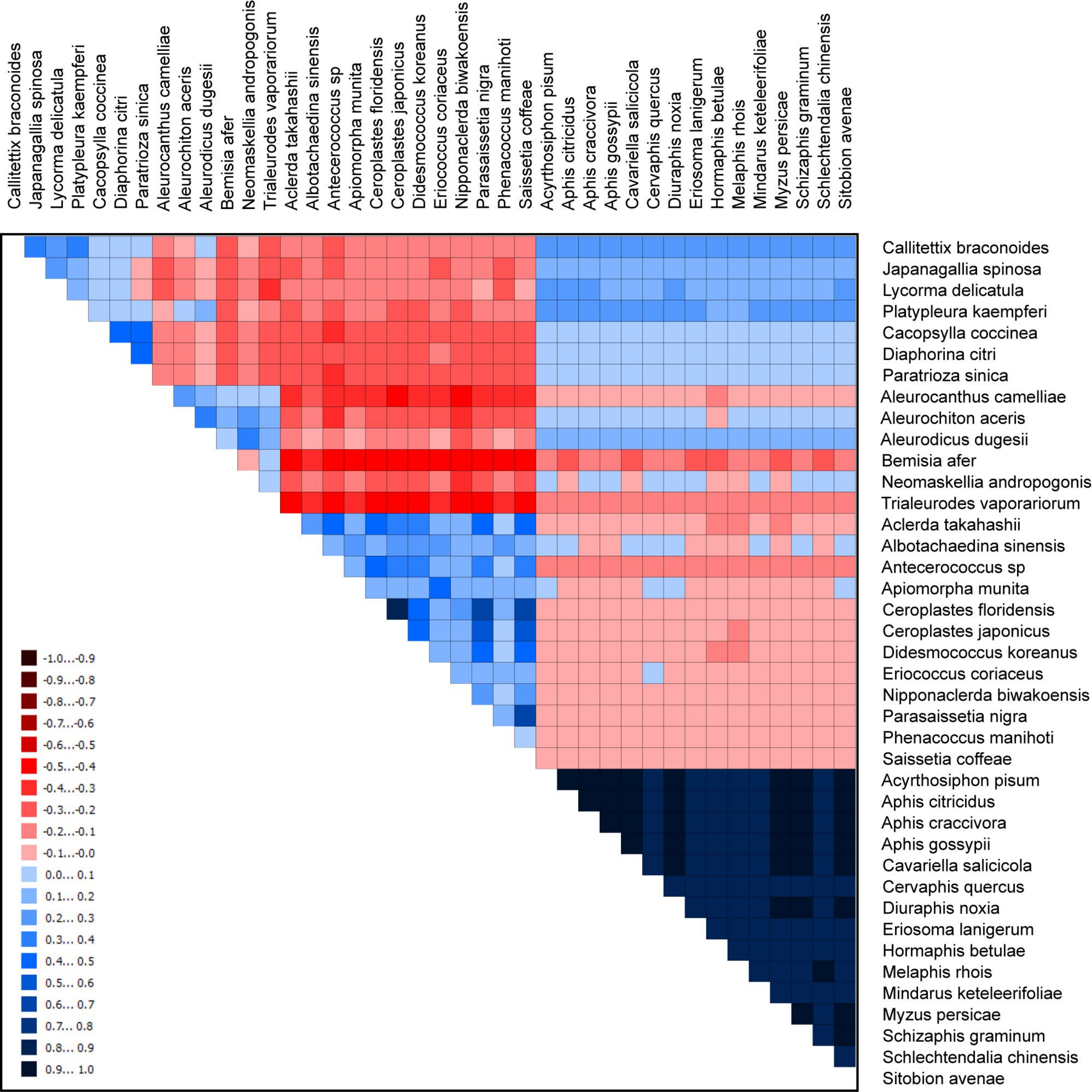

Callitettix braconoides
Japanagallia spinosa
Lycorma delicatula
Platypleura kaempferi
Cacopsylla coccinea
Diaphorina citri
Paratrioza sinica
Aleurocanthus camelliae
Aleurochiton aceris
Aleurodicus dugesii
Bemisia afer
Neomaskellia andropogonis
Trialeurodes vaporariorum
Aclerda takahashii
Albotachaedina sinensis
Antecerococcus sp
Apiomorpha munita
Ceroplastes floridensis
Ceroplastes japonicus
Didesmococcus koreanus
Eriococcus coriaceus
Nipponaclerda biwakoensis
Parasaissetia nigra
Phenacoccus manihoti
Saissetia coffeae
Acyrthosiphon pisum
Aphis citricidus
Aphis craccivora
Aphis gossypii
Cavariella salicicola
Cervaphis quercus
Diuraphis noxia
Eriosoma lanigerum
Hormaphis betulae
Melaphis rhois
Mindarus keteleerifoliae
Myzus persicae
Schizaphis graminum
Schlechtendalia chinensis
Sitobion avenae
-1.0...-0.9
-0.9...-0.8
-0.8...-0.7
-0.7...-0.6
-0.6...-0.5
-0.5...-0.4
-0.4...-0.3
-0.3...-0.2
-0.2...-0.1
-0.1...-0.0
0.0... 0.1
0.1... 0.2
0.2... 0.3
0.3... 0.4
0.4... 0.5
0.5... 0.6
0.6... 0.7
0.7... 0.8
0.8... 0.9
0.9... 1.0
